# Maternal Trauma and Psychopathology Symptoms Affect Refugee Children’s Mental Health But Not Their Emotion Processing

**DOI:** 10.1007/s10802-024-01182-0

**Published:** 2024-03-02

**Authors:** Julia E. Michalek, Lina Qtaishat, Sophie von Stumm, Amal El Kharouf, Rana Dajani, Kristin Hadfield, Isabelle Mareschal

**Affiliations:** 1 Youth Resilience Unit, Centre for Psychiatry and Mental Health, Wolfson Institute of Population Health, Queen Mary University of London, London, UK; 2 Taghyeer, Amman, Jordan; 3 Department of Education, University of York, York, UK; 4 Centre for Women Studies, University of Jordan, Amman, Jordan; 5 Department of Biology and Biotechnology, Faculty of Science, The Hashemite University, Zarqa, Jordan; 6 Trinity Centre for Global Health, School of Psychology, Trinity College Dublin, Dublin, Ireland; 7 School of Biological and Behavioural Sciences, Department of Psychology, Queen Mary University of London, London, UK

**Keywords:** Emotion processing, Refugee children, Mental health, War trauma

## Abstract

Refugee children’s development may be affected by their parents’ war-related trauma exposure and psychopathology symptoms across a range of cognitive and affective domains, but the processes involved in this transmission are poorly understood. Here, we investigated the impact of refugee mothers’ trauma exposure and mental health on their children’s mental health and attention biases to emotional expressions. In our sample of 324 Syrian refugee mother-child dyads living in Jordan (children’s *M*_*age*_=6.32, *SD* = 1.18; 50% female), mothers reported on their symptoms of anxiety and depression, and on their children’s internalising, externalising, and attention problems. A subset of mothers reported their trauma exposure (*n* = 133) and PTSD symptoms (*n* = 124). We examined emotion processing in the dyads using a standard dot-probe task measuring their attention allocation to facial expressions of anger and sadness. Maternal trauma and PTSD symptoms were linked to child internalising and attention problems, while maternal anxiety and depression symptoms were associated with child internalising, externalising, and attention problems. Mothers and children were hypervigilant towards expressions of anger, but surprisingly, mother and child biases were not correlated with each other. The attentional biases to emotional faces were also not linked to psychopathology risk in the dyads. Our findings highlight the importance of refugee mothers’ trauma exposure and psychopathology on their children’s wellbeing. The results also suggest a dissociation between the mechanisms underlying mental health and those involved in attention to emotional faces, and that intergenerational transmission of mental health problems might involve mechanisms other than attentional processes relating to emotional expressions.

**Supplementary Information:**

The online version contains supplementary material available at 10.1007/s10802-024-01182-0.

Childhood adversity is one of the main risk factors for affective disorders and related emotion processing biases. These biases usually reflect atypical and maladaptive processing of emotional information that can result in e.g., heightened threat vigilance, altered attention to emotional stimuli, impaired emotion recognition, and emotion dysregulation (e.g., Bodenschatz et al., 2019; Briggs-Gowan et al., 2015; Harms et al., 2019; Lakshman et al., 2020; McCoy et al., 2016; Nelson et al., 2020; Pollak, 2015a). Such atypicalities in emotion processing might arise from early experiences of threatening and unstable environments and contribute to the development of poor cognition and psychopathology (e.g., Pollak, 2015a). Caregiver trauma and mental health problems are also risk factors for poor child psychosocial functioning (e.g., Mirzaaghasi et al., 2014). As a result of the growing number of conflicts and security threats in the world, there are currently 110 million forcibly displaced people worldwide, over 43 million of whom are children (UNHCR, 2023). Displaced adults and children suffer the consequences of war trauma exposure and face post-displacement difficulties, which often result in increased risk of mental illness (Cratsley et al., 2021; McEwen et al., 2023). Notwithstanding the vast number of children affected by armed conflict, the consequences of these experiences on children’s wellbeing and emotion processing are still poorly understood.

Refugee children face a plethora of displacement-related stressors which place them at risk for emotional and behavioural problems, disruptions in emotion regulation and recognition, enhanced attention to threat, and overall poorer emotion processing (Burkhouse & Kujawa, 2023; Durà-Vilà et al., 2012; Gredebäck et al., 2021a; Hodes & Vostanis, 2018; Khamis, 2019; Michalek et al., 2022; Scherer et al., 2020; Yayan et al., 2020, but see Michalek et al., 2023). Whilst these socio-emotional impairments can occur as a direct result of war exposure, displacement, and continuing adversity, parental trauma and mental health problems are also likely to influence refugee children’s development (Miller & Rasmussen, 2017). Indeed, it has been shown that caregiver trauma and psychopathology affect children’s emotional and behavioural outcomes in non-refugee populations (Clavarino et al., 2010; Goodman et al., 2011; Lambert et al., 2014; Mirzaaghasi et al., 2014; Morris et al., 2012), and emerging research suggests similar effects in refugee families. Studies in post-conflict areas, war zones, and refugee settings highlight strong associations between child and caregivers’ distress, PTSD symptoms, and internalising and externalising problems (Betancourt et al., 2012, 2015; Field et al., 2013; McEwen et al., 2023; Meyer et al., 2017; Thabet et al., 2009). For example, Syrian refugee parents’ poorer mental health predicted their children’s emotional and behavioural problems (Eruyar et al., 2018), whilst parental war trauma exposure has been linked to child conduct problems and hyperactivity in refugee families (Bryant et al., 2018; Eruyar et al., 2018). Interestingly, parenting styles and parental displays of anger (often resulting from their own experiences of trauma and PTSD) also play a role in this context (Hinton et al., 2009; Sim et al., 2018; Thabet et al., 2009). For instance, maternal PTSD predicted children’s poorer identification of emotional expressions amongst Syrian refugees, with children’s impaired emotion recognition linked to harsher parenting (Gredebäck et al., 2021a; Peltonen et al., 2022). A recent meta-analysis reported that parental war-related trauma was linked to harsher parenting styles, which in turn mediated the association between parental trauma and child adjustment, including emotional symptoms, social problems, and quality of life (Eltanamly et al., 2021). On the other hand, higher levels of family acceptance and lower levels of community stigma were linked to fewer internalising problems among adolescents living in post-conflict Sierra Leone (Betancourt et al., 2015). Similarly, Syrian children and adolescents, living in Jordan and Lebanon, who reported higher family cohesion and lower levels of family conflict also reported fewer internalising and externalising problems, indicating that positive family environment is linked to higher mental wellbeing in refugee youth (Khamis, 2021). Few studies so far have focused on emotional processing biases in refugee children (e.g., Gredebäck et al., 2021b; Michalek et al., 2022, 2023) but research with non-refugee children suggests that such biases might be instrumental in linking parental and child mental health. Taken together, parental trauma, mental illness, poor parenting strategies, and unstable family environments may jointly contribute to impairments in emotional, cognitive, and behavioural outcomes in refugee children, although little is known about the mechanisms underlying these effects.

According to integrative models of familial risk for psychopathology, cognitive patterns exhibited by depressed or anxious parents (e.g., enhanced threat vigilance, negative affect, interpreting ambiguous situations as negative) might be a mechanism of intergenerational transmission of anxiety and depression (Bögels & Brechman-Toussaint, 2006; Goodman et al., 2011; Goodman & Gotlib, 1999; Hadwin & Field, 2010). Caregivers’ displays of atypical emotion processing could result in their children developing similar emotion processing biases, and in turn, symptoms of affective disorders. For instance, maternal anxiety levels have been linked to heightened attention to threat in infants and children (Aktar et al., 2019; Morales et al., 2017), and children of depressed mothers were found to misattribute sadness to other emotions (Kluczniok et al., 2016), display an attention bias to sad stimuli (Owens et al., 2016), perceive more negative affect in maternal emotional states (Luebbe et al., 2013), and display impaired emotion recognition overall (Priel et al., 2020). The link between caregiver-child emotion processing, however, is less clear, with some studies suggesting that maternal biases are predictive of their children’s biases (de Lijster et al., 2020; Waters et al., 2015) and others finding no relationship (Aktar et al., 2019; Platt et al., 2021), highlighting the complexity of the cognitive mechanisms involved in familial psychopathology transmission. Taken together, it is possible that the cognitive biases displayed by caregivers, combined with changes in family dynamics and negative parenting strategies which show associations with maladaptive emotion regulation, might exacerbate mental health symptoms, behavioural problems, and emotion processing disturbances in their children.

Although the effects of refugee parental trauma and psychopathology on their children’s psychosocial functioning are well-established (e.g., Bryant et al., 2018), the mechanisms behind this potential transmission of risk in the refugee context are not well understood. With multiple war- and displacement-related difficulties faced by refugees, it is imperative to understand how exposure to chronic adversity impacts children’s mental health and their development of emotional function. To address this gap in the literature, we investigated the association between attention biases to emotional expressions in Syrian refugee mother-child dyads and explored if maternal war-related trauma and psychopathology symptoms is linked to child attention biases to facial expressions and their mental health. Based on previous research we hypothesised that (1) maternal trauma and mental health symptoms would be related to their child’s mental health outcomes, (2) mothers and their children would display attentional biases towards both angry and sad facial stimuli, and (3) mother and child attention biases would be positively correlated. We also hypothesised that (4) mother trauma/mental health scores would relate to both the mother’s and her child’s attention biases, and that children’s attention biases would correlate with their mental health outcomes. Previous studies with Syrian refugee children identified an attentional bias towards threat (i.e., increased sustained attention to angry facial expressions) that was linked to war-related trauma exposure, suggesting children might face difficulties with disengaging from a threat-relevant stimuli, rather than exhibit threat hypervigilance (Michalek et al., 2022). Therefore, we tested here whether an attention bias to angry expressions reflected a hypervigilance to anger or difficulties disengaging from anger in our sample of mother-child dyads.

## Methods

### Study Setting

Since the beginning of the Syrian civil war in 2011, over 12 million Syrians have been forcibly displaced (UNHCR, 2023), many fleeing to neighbouring countries – Turkey, Lebanon, and Jordan (UNHCR, 2021b). Jordan currently hosts around 700,000 Syrian refugees, over 80% of whom live in urban areas in the Governorate of Amman and Mafraq (UNHCR, 2021a). In addition to the traumatic experiences of war and displacement, life after resettlement poses substantial challenges, including poverty, limited or lack of access to employment or schooling, and having to navigate foreign systems and customs (e.g., Hall, 2022). Further, the economic impact of the refugee crisis on Jordan has important consequences for the livelihoods of Syrian refugee families. The pre-existing pressures on the public healthcare system and education, fragile economy, and resources constraints were greatly exacerbated during the refugee crisis, resulting in many refugees living in Jordan struggling with basic needs and access to education, facing debt, as well as shelter and food insecurities (Hadfield et al., 2022; Hall, 2022; Tiltnes et al., 2019), all of which have important consequences on refugee children’s wellbeing and emotional development.

### Participants

Participants were Syrian refugee mother–child dyads living in Jordan (*N* = 324, child *M*_*age*_ = 6.32 (1.18), 50% female, maternal *M*_*age*_ = 32.61 (7.02)). Data collection took place at participants’ homes in Amman (*n* = 235) and in the Al-Zaatari refugee camp (*n* = 86). Families living in Amman and Al-Zaatari did not significantly differ on child or maternal age, child gender distribution, attention task, or mental health outcomes (all *p* > .05). However the families living in Al-Zaatari were more impoverished than families living in Amman (*t*(216.47) = 4.06, *p* < .001, Cohen’s *d* = 0.44).

Refugee children and their mothers were recruited through the non-profit organisation, *Taghyeer*, to participate in a randomised controlled trial (RCT) evaluation of the “We Love Reading” (WLR) programme for children (FIERCE). Child attention biases were measured at T1, prior to the reading program (February–May 2021), whereas maternal attention biases, trauma exposure, and mental health measures were collected at T2, after the reading program (May–August 2021; Appendix S1). WLR is a non-dialogic shared book reading programme, whose aim is to foster children’s love of reading (Dajani, 2019). Members of the local community (mostly mothers) conduct once-a-week sessions to read aloud to local children (6–10 children per group) from a set of books they received from the WLR organisation. The RCT evaluated the effects of the reading programme on children’s literacy and reading attitudes (https://osf.io/gcv5z/). Although the reading programme was previously found to have small, short-term effects on the recognition of certain emotions in children (Michalek et al., 2021), we did not expect it to influence their mothers’ outcomes (from whom measures were collected after the programme). This is because WLR programme’s focus is on improving children’s attitudes to reading and the RCT evaluated its effects on reading acquisition and literacy. Since WLR is designed for children, we had no reason to expect that it would affect their mothers’ cognitive or affective processing or wellbeing; nonetheless, we conducted a sensitivity analysis controlling for the RCT groups (control v experimental) for all our variables of interest.

### Ethical Considerations

The project was granted ethical approval from the Trinity College Dublin research ethics board (01E/2020/10) and the Prime Minister’s Office of Jordan. Mothers gave their written consent and children their assent prior to taking part in the study. Families were reimbursed 5JD for their participation at timepoint 1 (February – May) and 10JD at timepoint 2 (May – August). Data were analysed using R (R Core Team, 2020). Study data and analysis code are publicly available on the study OSF page (https://osf.io/gcv5z/).

### Data Collection Procedure

Demographic data, maternal trauma exposure, and mother and child psychopathology were measured using questionnaires originally developed in Arabic or adapted to Arabic, administered by native Arabic-speaking field workers. Mothers and their children were tested in their homes. All questionnaire measures (mother and child) were collected orally from the mothers using Kobo Toolbox (2021). Detailed information of all questionnaire measures is presented in the Supporting Information Appendix S2.

### Maternal Trauma and Mental Health

Maternal trauma was measured with the self-reported Traumatic Events Checklist (TEC, Panter-Brick et al., 2009), consisting of 21 *yes/no* questions related to war trauma and displacement. Due to the distressing nature of the trauma questionnaire, only a subset of participating mothers completed this measure before it was replaced with the less upsetting PTSD questionnaire: the PTSD Checklist for DSM-5 (PCL-5, Weathers et al., 2013) which includes 20 items (Cronbach’s α = 0.92). This resulted in two subsets of mothers: those with trauma scores (*n* = 133) and those with PTSD symptoms scores (*n* = 124). The anxiety subscale of the short form Depression, Anxiety, Stress (DASS-21, Henry & Crawford, 2005) was used to measure maternal anxiety symptoms (7 items, Cronbach’s α = 0.79). Finally, maternal depression symptoms were measured using the Centre for Epidemiological Studies – Depression Scale (CES-D, Radloff, 1977), which includes 10 items (Cronbach’s α = 0.79).

### Child Mental Health

Child mental health was reported by their mothers using the short Arabic version of the Paediatric Symptoms Checklist (PSC–17, Jellinek et al., 1988). PSC-17 contains three subscales which measure separate constructs: internalising (5 items, Cronbach’s α = 0.59), externalising (7 items, Cronbach’s α = 0.74), and attention problems (5 item, Cronbach’s α = 0.65).

### Attention Bias

We used a standard dot probe task (Macleod et al., 1986) to measure the attention biases to angry and sad facial expressions, using photographs of 20 adult actors (10 female) displaying 4 emotional expressions: anger, sadness, happiness, and neutral taken from the Radboud Faces Database (Langner et al., 2010). Happy expressions were only used for the practice trials (*n* = 12) shown to the children only and were not part of the experiment. Neutral faces were paired with the same identity emotional face. The images were validated for emotion recognition accuracy (via a standard emotion identification task) by a subset of the participants (children *n* = 14, mothers *n* = 14). All emotional expressions had good recognition accuracy among children and mothers: angry = 94%, sad = 77%, neutral = 60%.

**Fig. 1:**
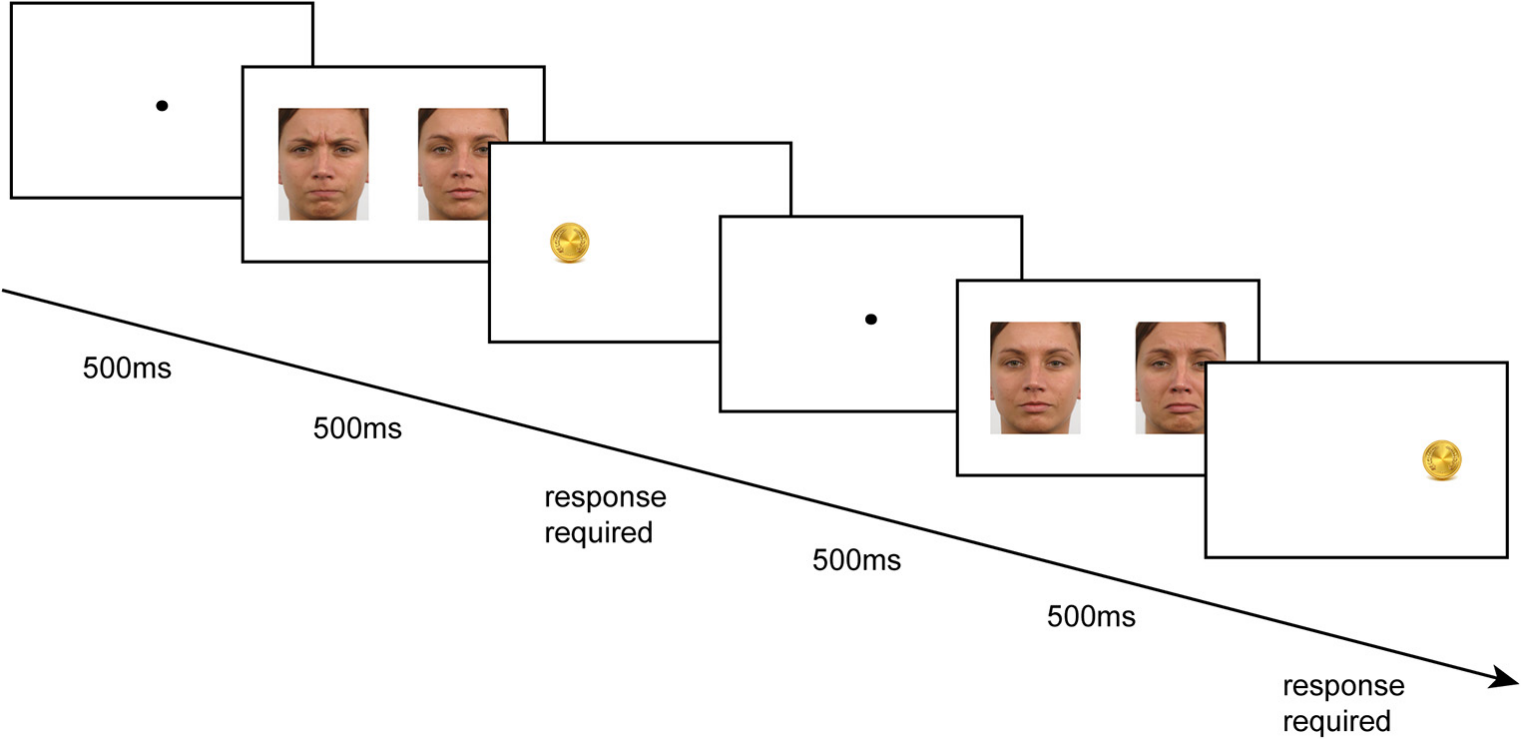
Examples of two congruent trials with stimuli pairs angry-neutral and sad-neutral. Participants should press either the *left* or *right* arrow depending on the coin location

The task ran in Matlab (Mathworks, 2020a) and Psychtoolbox (Brainard, 1997). Angry and sad expressions were always shown in a pair with the same identity neutral face (*n* = 40 trials each for the angry/neutral and the sad/neutral pairs). We also used 20 baseline trials with pairs of (same identity) neutral–neutral expressions. Stimuli were displayed in colour on a Dell laptop computer (screen size = 27.7 cm × 15.6 cm, screen resolution = 1366 × 768). At the viewing distance of 57 cm, images of the emotional faces subtended 7.1 × 9.5 degrees of visual angle and the distance between the centre of the screen and the centre of each image was 5.8 degrees of visual angle. All stimuli were randomly interleaved across the 100 trials. Each trial began with a fixation dot for 500ms, followed by the face pair displayed for 500ms. Upon extinction of the face pair, a coin (probe) appeared in the location of either the emotional (congruent) or neutral (incongruent) face (Fig. 1). Congruency and emotional expression locations (left or right) were evenly split and randomised across trials. Participants indicated the position of the coin (left or right) as quickly and accurately as possible using a keypress, and the probe remained on the screen until a response was made. Participants were instructed to ‘catch’ as many coins as possible. After each block of 40 trials and at the end of the experiment participants were informed of the number of coins they had collected. To measure attention biases, we calculated accuracy (% correct responses to the probe location) and reaction times for the correct congruent and incongruent trials, separately for the two emotional expressions (angry and sad). We also calculated accuracy and reaction times for the baseline trials where both faces were neutral. Faster reaction times on congruent trials (where probe is located on the same side as the emotional face) suggest facilitated attention to emotional expressions. The attention bias for the two emotion categories was calculated by subtracting reaction times on congruent trials from reaction times on incongruent trials.

A total of 317 mothers and 322 children completed the dot probe task. A subset of participants (children *n* = 33, mothers *n* = 12) who did not understand instructions, did not pay attention to the rules, or were distracted, were excluded from all analyses of attention biases. Further, participants with accuracy less than 65% (children *n* = 25, mothers *n* = 7), and individual trials with reaction times faster than 200ms (suggesting responses not following the task instructions), slower than 7s, or more than 3 standard deviations away from individual participants’ mean reaction time (< 1% of trials) were excluded from the analysis, in line with previous studies (e.g., Briggs-Gowan et al., 2015). This resulted in a final sample of 298 mothers and 264 children (Table S2).

### Statistical Analysis

There were no effects of child age or gender, nor mother age on either the angry or sad attention bias (*p* > .05). To establish if participants were significantly biased in their attention towards the emotional faces, we conducted one-sample t-tests separately for each bias.

In addition to the main analyses on attention biases, we also conducted exploratory analyses to differentiate *vigilance* and *disengagement difficulties* from the significant attention bias measures following Koster et al. (2004). The exploratory analyses used participants’ average reaction times (RTs) to the neutral–neutral trials as a baseline measure of their attention to the task when no emotional stimuli are present. The congruent trials refer to emotional trials (i.e., angry–neutral or sad-neutral) where the probe appears in the same location as the emotional stimulus, and the incongruent trials refer to emotional trials where the probe appears in the opposite location to the emotional stimulus. Using the congruent, incongruent, and *baseline* RTs measures, the following analyses were conducted:

1. *Vigilance*: Shorter RTs on (emotional) congruent trials compared to baseline RTs indicate heightened vigilance to emotional stimuli, because participants’ gaze and attention remains in the location of the emotional stimulus. Therefore, they respond more quickly when the probe is located behind the emotional face, compared to their baseline response time.
2. *Disengagement difficulties*: Longer RTs on (emotional) incongruent trials compared to baseline RTs indicate difficulties in disengagement from emotional stimulus, because participants require more time to switch their attention away from an emotional stimulus, compared to their baseline response time.

Paired samples t-tests were conducted to compare congruent RTs to baseline RTs (*vigilance*) and to compare incongruent RTs to baseline RTs (*disengagement difficulties*) for mothers and children separately. Overall, compared to baseline RTs, shorter congruent RTs indicate heightened vigilance, while longer incongruent RTs indicate disengagement difficulties.

To establish the relationship between maternal and child attention biases, we conducted two linear regressions, with maternal attention bias as predictor and child attention bias as outcome, for anger and sadness separately. To test the association between maternal questionnaire measures and child attention biases, a series of separate, simple linear regression models was conducted with maternal trauma, PTSD, anxiety, and depression as predictors and child angry and sad attention bias as outcomes (4 tests for each emotion bias). We used twelve simple linear regressions to investigate the association between maternal trauma and mental health scores (predictors) and child mental health scores (outcomes). As the maternal trauma exposure and PTSD symptoms were only collected from different subsets of participating mothers, we conducted separate linear regressions for each maternal predictor. To account for multiple testing all regression models were corrected using a Holm-Bonferroni method (Holm, 1979).

Prior to the main analyses, we conducted a sensitivity analysis for any potential effects of the reading programme on our measures of interest. There were no significant differences between the experimental and control groups in child or mother attentional biases or mental health outcomes (all *p* > .100, Table S7), but mothers who took part in the reading programme reported experiencing a higher number of traumatic events than mothers in the waiting control group (*t*(129.72) = -3.91, *p* < .001, *d* = -0.68). To account for any potential effects of reading programme participation, we reran our linear regression models controlling for experimental versus wait-listed control condition. These sensitivity analyses showed that reading programme participation condition did not affect our findings; corresponding results are reported in full in the supplementary materials (Tables S8–S11).

## Results

### Participant Demographics, Trauma Exposure, and Mental Health

**Fig. 2:**
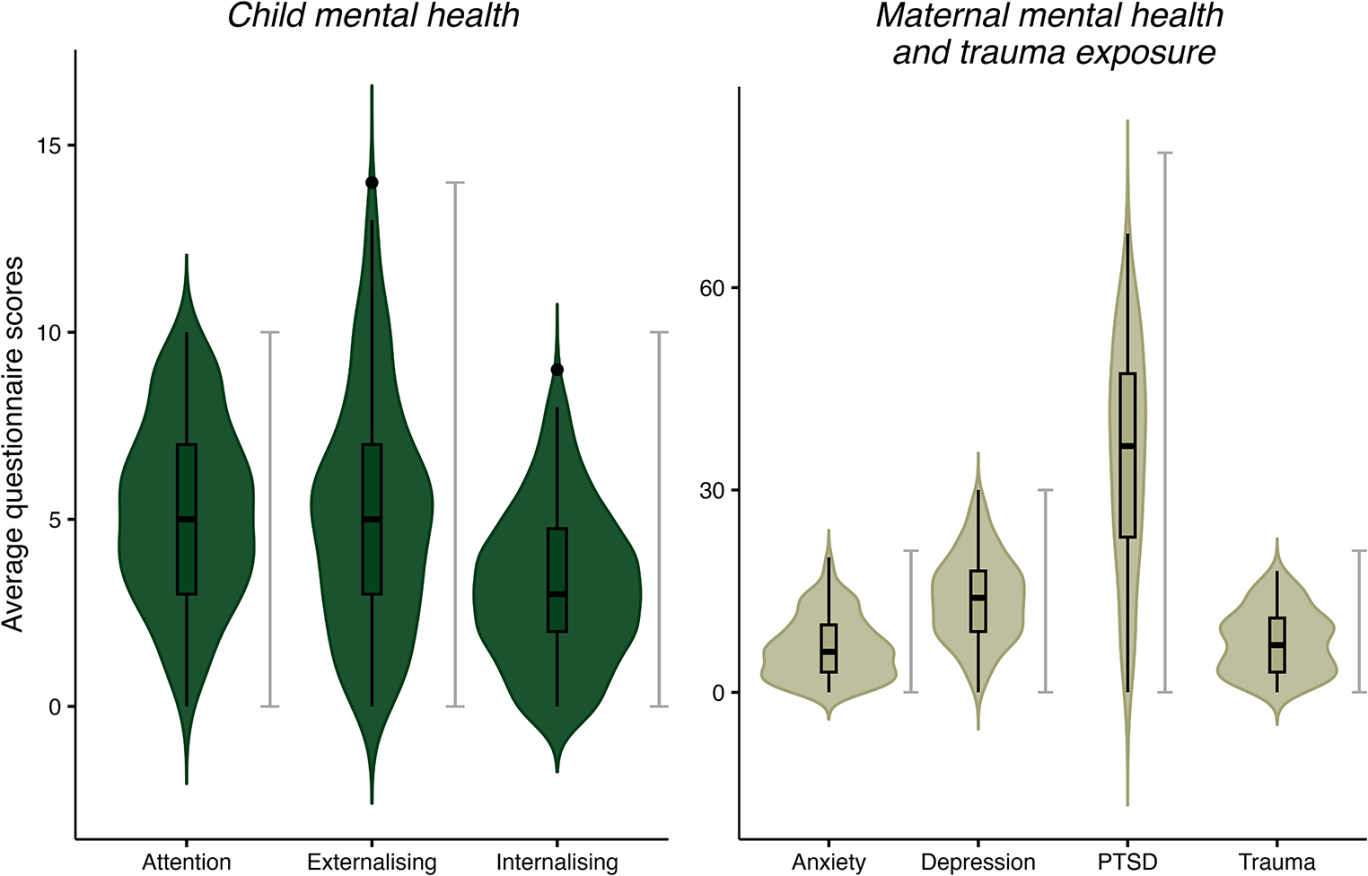
Child mental health *(left)*, and maternal trauma exposure and mental health *(right)* scores. Trauma = TEC (Traumatic Events Checklist), PTSD symptoms = PCL-5 (PTSD Checklist for DSM-5), Anxiety symptoms = DASS-Anxiety (Anxiety subscale of the Depression, Anxiety, Stress Scale short form), Depression symptoms = CES-Depression (Centre for Epidemiological Studies – Depression Scale), Paediatric Symptoms Checklist (PSC-17) measures all child mental health symptoms. Grey bars indicate possible range for each measure

Demographics, trauma, and mental health scores are presented in Fig. 2 and Tables S11 and S2. Children’s age ranged between 4 and 9 years old (*M =* 6.32 years, *SD* = 1.18). 317 of the participating families reported on the amount of time they spent in Jordan. As most families fled Syria between 2011 and 2014, the majority of children in our sample were born in displacement (children born in Syria *n* = 56; born in Jordan *n* = 261). Participating children had spent on average 77% of their lives in Jordan (*SD* = 17.52%), ranging between 14% and 100%. Linear regression analyses showed that the proportion of life spent away from Syria did not significantly relate to child anger or sadness attention bias, nor to child internalising, externalising, or attention problems (all *p* > .400). Similarly, there were no differences in child emotion processing or mental health measures between the children who were born in Syria compared to children who were born in Jordan (all *p* > .100). Child mental health scores reported by the mother were in the low range of the scales, indicating good mental health (as per cut-off scores established in non-refugee samples; Jellinek et al., 1988, although we note that mental health cut-offs established in Western non-refugee populations may not be valid for this Syrian refugee sample, e.g., Wells et al., 2015).

Maternal trauma and mental health measures were all significantly correlated (Table S3), and mothers reported experiencing 7.52 traumatic events on average (*SD* = 4.69) out of a possible 20, with almost 80% reporting having felt that their life was in danger. Over 50% of the responding mothers reported having lived in a refugee camp, being forcibly separated from their family, and witnessing other extreme war-related events, such as bombardments and seeing a wounded or dead body. The details of the traumatic events experienced by the refugee mothers (as questions included in the TEC) are presented in Fig. S1. Mothers reported high scores for PTSD and depression symptoms, indicating potential mental health problems in these two domains (Blevins et al., 2015; Radloff, 1977; Weathers et al., 2013), but their anxiety symptoms were not indicative of anxiety issues (Henry & Crawford, 2005).

### Effects of Maternal Trauma and Mental Health on Child Mental Health

In a series of simple linear regressions we found that maternal trauma exposure was significantly linked to child internalising and attention problems, but not to externalising problems. Maternal anxiety, depression, and PTSD symptoms were all associated with child internalising, externalising, and attention problems (Table 1, Fig. S2). All results remained significant after correcting for multiple comparisons, apart from the association of maternal PTSD symptoms with child externalising problems (*p* > .05).

**Table 1:** Twelve simple linear regression models of the effects of maternal trauma and mental health on child mental health

Maternal predictors	F	df	p	adj.R^2^	t	b	β
*Child internalising problems*							
Trauma exposure	13.56	1, 131	**< 0.001**	0.09	3.68	0.14	0.31
PTSD	9.38	1, 121	**0.003**	0.06	3.06	0.03	0.27
Anxiety	14.46	1, 284	**< 0.001**	0.05	3.80	0.10	0.22
Depression	53.58	1, 279	**< 0.001**	0.16	7.32	0.13	0.40
*Child externalising problems*							
Trauma exposure	1.09	1, 128	0.299	< 0.01	1.04	0.06	0.09
PTSD	4.14	1, 122	0.044	0.03	2.03	2.03	0.18
Anxiety	21.77	1, 281	**< 0.001**	0.07	4.67	0.18	0.27
Depression	19.29	1, 276	**< 0.001**	0.06	4.39	0.12	0.26
*Child attention problems*							
Trauma exposure	6.73	1, 131	**0.011**	0.04	2.59	0.11	0.22
PTSD	15.67	1, 120	**< 0.001**	0.11	3.96	0.05	0.34
Anxiety	16.83	1, 283	**< 0.001**	0.05	4.10	0.12	0.24
Depression	26.69	1, 279	**< 0.001**	0.09	5.17	0.11	0.30

Note Regressions were conducted separately for each maternal predictor due to the trauma exposure and PTSD symptoms measure having been collected from two subsets of the participating mothers. Associations which remained significant after Bonferroni corrections are presented in bold. *b* = unstandardised estimate, *β* = standardised estimate

### Attention Bias

Outliers > 3SD from the group mean were excluded from the analysis (children: angry bias *n* = 7, sad bias *n* = 5; mothers: angry bias *n* = 3, sad bias *n* = 5). Children and mothers performed the task well, with 94% and 99% accuracy respectively, and average reaction times of 1110 ms for the children and 580 ms for the mothers (Table S4).

**Fig. 3:**
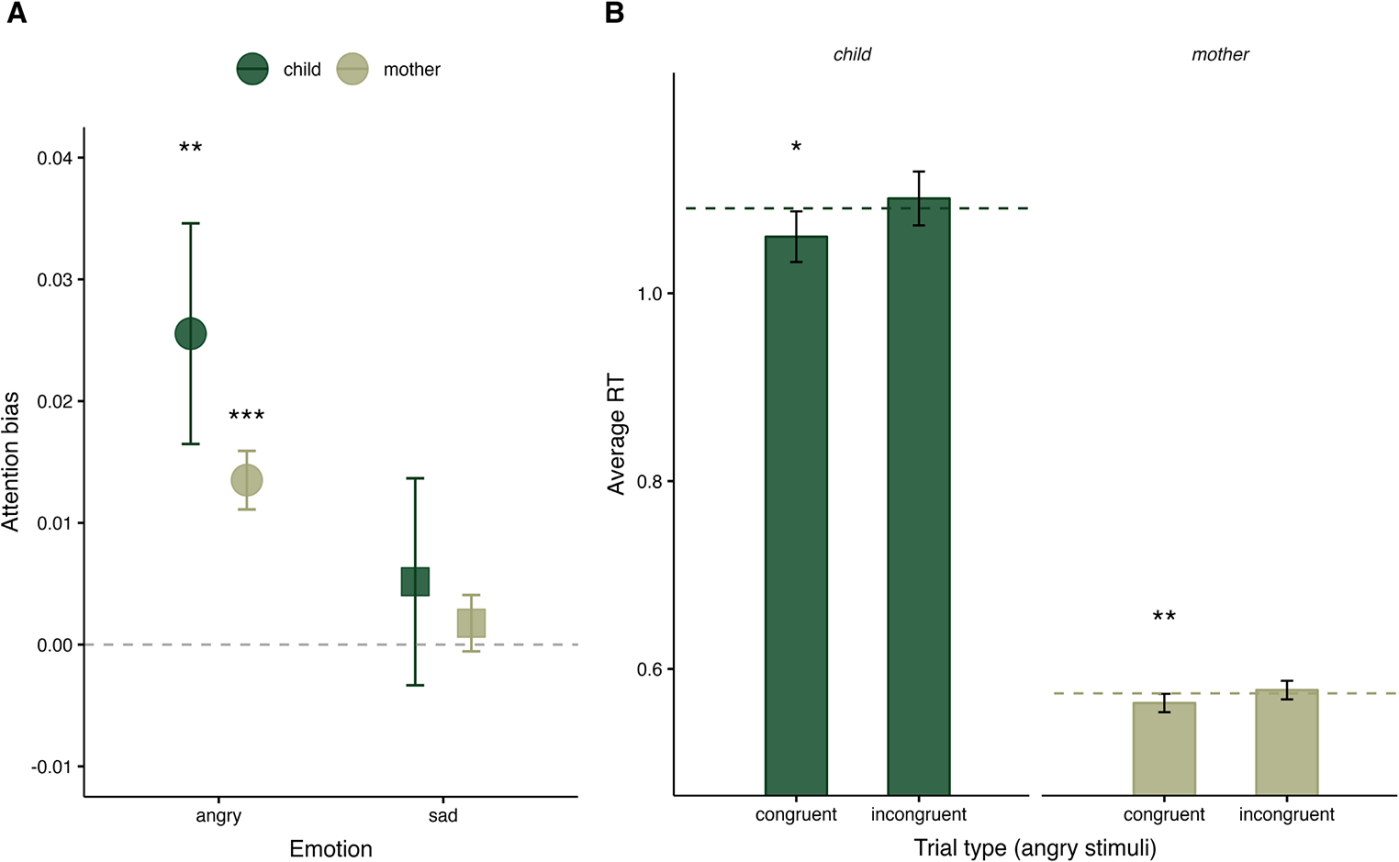
(**A**) Average attention biases for *angry* (*circles*) and *sad* (*squares*) stimuli for children *(green)* and mothers *(grey)*. Error bars represent standard error of the mean. (**B**) Average reaction times *(RT)* in seconds for each trial type for the anger stimuli only. Horizontal dashed lines represent baseline reaction times to neutral faces (neutral-neutral trials), error bars represent 95% confidence intervals. *p < .05, **p < .01 and ***p < .001

Both children, *t*(256) = 2.82, *p* = .005, and their mothers, *t*(294) = 5.62, *p* < .001, had a significant attention bias towards angry expressions, with small and moderate effect sizes (Cohen’s *d* = 0.18 and *d* = 0.33, respectively). Neither the children, *t*(258) = 0.61, *p* = .544, Cohen’s *d* = 0.04, nor their mothers, *t*(292) = 0.76, *p* = .447, Cohen’s *d* = 0.04, displayed a significant attention bias towards sad expressions (Fig. 3A).

### Threat Vigilance or Disengagement Difficulties?

Following Koster et al. (2004), we investigated whether the anger bias reflected a heightened vigilance to threat or disengagement difficulties. We found that RTs to congruent angry trials were significantly shorter than baseline RTs in children *t*(259) = -2.05, *p* = .041, Cohen’s *d* = − 0.13, and mothers, RTs, *t*(293) = -2.90, *p* = .004, Cohen’s *d* = − 0.17, indicating that the anger bias reflects a heightened vigilance to threat. The difference between RTs on incongruent angry trials and baseline RTs did not reach statistical significance in either the children, *t*(259) = 0.81, *p* = .418, Cohen’s *d* = 0.05, or their mothers, *t*(293) = 1.01, *p* = .312, Cohen’s *d* = 0.06, suggesting no disengagement difficulties (Fig. 3B).

### Attention Biases and the Effects of Trauma and Mental Health

Contrary to our third hypothesis, we found that mothers’ and children’s attention biases were not related for angry, *F*(1, 230) = 0.80, *p* = .37, *R*^*2*^ < − 0.01, or sad stimuli, *F*(1, 231) = 0.02, *p* = .89, *R*^*2*^ < − 0.01 (Fig. S3). Further, contrary to our fourth hypothesis, we found no significant associations of maternal trauma exposure, PTSD, anxiety, or depression symptoms with either maternal or child attention biases. There was also no significant links between child mental health problems and child’s attention biases (all *p* > .3, see Table S5).

## Discussion

In our study, we examined refugee mother-child mental health and whether attention biases towards emotional facial expressions in mother-child dyads were associated with each other or with psychopathology symptoms. As expected, we found that mothers’ trauma and mental health were related to their child’s mental health. We also found that refugee mothers and their children displayed threat hypervigilance, although we found no correlation between mother and child attention biases, and attention biases were also unrelated to maternal trauma exposure, or maternal or child mental health.

Our findings add to the growing body of research highlighting the importance of maternal trauma exposure and mental health on their child’s development in a refugee context (e.g., Bryant et al., 2018; Eruyar et al., 2018; McEwen et al., 2023). Overall, mothers reported high levels of trauma exposure, PTSD, and depression symptoms, indicative of potential heightened psychopathology risk (Radloff, 1977; Weatheres et al., 2013), however their children’s internalising, externalising, and attention problems were suggestive of lower psychopathology risk in this sample (based on cut-off scores from previous studies in non-refugee samples, e.g., Jellinek et al., 1988). Mental health difficulties and problematic behaviours amongst refugee children are reportedly linked to increased traumatic experiences (Karadag & Ogutlu, 2021; Yayan et al., 2020), and to poorer parental mental health (Bryant et al., 2018; Meyer et al., 2017). We found here that maternal depression and anxiety symptoms were overall better predictors of their child’s mental health than maternal trauma and PTSD, with the latter only linked to child internalising and attention, but not externalising problems, contrary to previous reports (e.g., Bryant et al., 2018). It is possible that externalising or conduct problems in refugee children could depend more on other factors previously reported for non-refugee populations, such as negative parenting styles (Schulz-Heik et al., 2010; Viding et al., 2009). It is worth noting that the relatively low scores on psychopathology symptoms for the refugee children in the current study might be unique to the studied population, as previous findings suggest high prevalence of symptoms of PTSD, depression, and anxiety among refugee children (Henley & Robinson, 2011; McEwen et al., 2023; Panter-Brick et al., 2018a). Our results might be linked to the young age of the participating children, and the fact that the majority of them were born in displacement and would not have had any direct exposure to war. However, these findings might also hint at potential protective factors related to the family environment and contextual resilience (Panter-Brick, Hadfield, Panter-Brick et al., 2018a, b).

Both mothers and their children displayed an attention bias towards angry faces only, consistent with previous reports linking adversity with attention biases towards threat (e.g., Briggs-Gowan et al., 2015; Powers et al., 2019; Roy et al., 2008). The attention bias to anger reflected a heightened vigilance to threatening faces (albeit with small effect sizes), with no difficulties in disengaging from the stimulus. This is somewhat at odds with an earlier experiment we conducted with a separate group of Syrian refugee children, where we found that higher trauma in children was not linked to vigilance to threat (children showed initial avoidance of angry and happy expressions) but was related to an increased sustained attention to anger, suggesting difficulties disengaging from threat (Michalek et al., 2022). However, with substantial methodological differences between the two experiments it is difficult to directly compare these results. Indeed, in our previous experiment we measured attention using eye-tracking only and children were shown 4 different emotional faces at a time with no task other than to simply view the faces (‘free viewing’). Our dot probe task here is also confined to the initial stages of attention allocation since stimulus presentation was very short (500ms), whereas in the previous experiment we measured children’s eye movements (scan paths) over 4000ms. It is likely that being required to perform a task (specifically to give a speeded response) and a limited viewing time tap into slightly different cognitive mechanisms than simply free viewing images for an extended period.

Our results are consistent with a growing body of work reporting hypervigilance to threat in children who are at risk for poorer mental health following early adversity (e.g., Briggs-Gowan et al., 2015; Pollak et al., 2001; Roy et al., 2008), suggesting that heightened initial detection of threat might be an important mechanism in the formation of detrimental cognitive patterns (Harms et al., 2019). Threat hypervigilance has been previously linked to adversity and psychopathology in non-refugee populations (Abend et al., 2018; Bar-Haim et al., 2007; Felmingham et al., 2011, although see Lisk et al., 2020) and could possibly reflect more general cognitive control difficulties (Cisler & Koster, 2010; Eysenck et al., 2007) and potential emotion regulation problems. Attention biases in refugee children could point to maladaptive cognitive strategies, which might lead to development of internalising and externalising symptoms in later life (Harms et al., 2019). Interventions or strategies which alter threat attention biases could represent a potential target to increase socio-emotional processing abilities and improve mental wellbeing in refugee children.

Surprisingly, we found that the attention biases to emotional expressions were largely unaffected by maternal trauma and mother/child psychopathology symptoms. These results differ from the previously well-established links between adversity, mental health, and attention biases, both in children and adults (e.g., Bodenschatz et al., 2019; Cisler & Koster, 2010; Hadwin et al., 2003; Pollak, 2015b; Reid et al., 2006), although recent meta-analyses and reviews suggest some inconsistencies (e.g., see Kruijt et al., 2019; Lisk et al., 2020). Threat hypervigilance might be reflective of the chronic stress of displacement and general life difficulties related to the refugee experience. Although it is surprising that this hypervigilance bias was not linked to poorer mental health here, the overall higher level of wellbeing and mental health of children in the current study might explain this lack of association.

We also did not find the expected associations between child attention biases and their mothers’ mental health. Previous studies of non-refugee populations show that children of depressed mothers usually display a biased attention to dysphoric stimuli (e.g., sad expressions), and children of anxious mothers display an enhanced attention to threat (Burkhouse et al., 2015; Kujawa et al., 2011; Morales et al., 2017; Owens et al., 2016). Interestingly, Gibb et al. (2022) found that maternal depression moderated children’s attention to sad faces, where younger children of depressed mothers displayed attentional avoidance while older children in this group displayed enhanced attention to sad stimuli. This hints at important developmental differences in the direction of attention biases in children at risk for depression and highlights potential developmental trajectory of emotion regulation atypicalities. In refugee populations, maternal PTSD symptoms have been previously linked to lower recognition accuracy of sad and happy emotions in their children (Gredebäck et al., 2021a). Yet, we found no evidence that affective attention biases were associated with the transmission of psychopathology risk, although the non-clinical nature of the mental health measures in our study, and a lack of control (non-refugee) group make comparisons difficult.

It is likely that trauma experienced (and reported) by the child affects the child’s emotional development more than trauma experienced by the mother. Children in the current study may be unaware of their mother’s traumatic experiences, and they are unlikely to have directly experienced war-related trauma themselves, as most children were born in displacement (82%). Considering the young age of the children, the length of displacement, and the current living conditions (mostly urban neighbourhoods) of the refugee families in our study, it is likely that the participants experienced other types of adversity not captured by our trauma measure, such as food insecurity, debt, unemployment, limited service provision, and lack of social support (e.g., Hall, 2022), and these post-displacement difficulties might have greater influence on children’s emotional processing than mothers’ trauma. Furthermore, maternal trauma and mental health itself might be less predictive of child facial emotion processing, with other family factors - such as parenting strategies and social support - playing a more important role, especially in humanitarian context (Peltonen et al., 2022). Caregiver mental health might influence refugee children’s development through parenting styles and parent-child relationship (e.g., Bryant et al., 2018; Eltanamly et al., 2021), rather than through cognitive biases. Programmes targeting positive parenting strategies, increasing family cohesion, and improving parent-child relationship through parenting training might be particularly beneficial for refugee children’s mental health and emotional development (Bosqui et al., 2022; Bryant et al., 2018; Eruyar et al., 2018; Khamis, 2021).

We found no relationship between mother and child attention biases for either angry or sad facial expressions. With few studies investigating these associations, and the variability of findings in the literature, our results are contrary to some reports (de Lijster et al., 2020; Waters et al., 2015) and in line with others (Aktar et al., 2019; Platt et al., 2021). It is possible that the higher variability in the distribution of children’s biases (average *SD* = 0.14) as compared to the biases of their mothers (average *SD* = 0.04) might blur the association. Whilst research in non-refugee populations largely supports the importance of emotion processing biases in the transmission of psychopathology risk within families (e.g., Kluczniok et al., 2016), our results suggest that other aspects of cognition and behaviour could play a more important role in this transmission in humanitarian settings. Including measures of mother’s *expressions* of anger and sadness in everyday life, rather than only their *perception* of or *attention* to these emotions might help clarify potential impact of their affective processing on emotion biases in their children, as mothers’ expressive styles and displays of facial affect have been shown to influence children’s emotion recognition and regulation in non-refugee populations (Camras et al., 1990; Nelson et al., 2012). Overall, our results suggest that maternal attention biases are not related to their child’s attention biases, and that transgenerational effects of familial psychopathology may be influenced by other factors, such as parenting or attachment styles (e.g., Thabet et al., 2009).

Our study has some limitations. Firstly, despite the dot probe task being widely used to study attention biases, it generally has poor test-retest reliability and poor internal consistency (Brown et al., 2014; Macleod et al., 2019; Schmukle, 2005; Staugaard, 2009; Xu et al., n.d.), although some have reported good internal consistency (e.g., Bar-Haim et al., 2007). Longitudinal attention bias data would be helpful in determining the consistency of emotion processing in our sample. Furthermore, as attention can shift within the first 200ms of stimuli presentation (Kappenman et al., 2014; Müller & Rabbitt, 1989), it is possible that children might disengage their attention between the presentation of the stimulus and the presentation of probe, although it is unclear if this very rapid shift of attention could occur in children. Secondly, since mothers reported on their child’s behaviours, it is possible that those mothers with worse mental health might have perceived their child’s mental health more negatively, and thereby reported higher child psychopathology. Parental emotional distress and anxiety levels have been shown to influence their reporting of their child’s anxiety (Krain & Kendall, 2000; Niditch & Varela, 2011), although parents are often thought to be reliable reporters of child mental health, with parental reports used across many studies (e.g., Abate et al., 2018; Murphy et al., 2012). It is also important to note that although our study is cross-sectional in design, the data from the mothers were collected approximately 3 months after the collection of the children’s measures. Given the stability of the families’ living conditions, we expected biases to remain stable, but this time difference might have contributed to the lack of associations between children’s and mothers’ attentional biases. Future studies should investigate mental health outcomes and emotion processing biases measured at multiple timepoints in both children and caregivers to examine developmental trajectories in emotional processing in refugee youth.

Taken together, our findings highlight the important effects of mother’s war trauma exposure and mental health on their children’s wellbeing. The attention bias displayed by mothers and children to angry faces reveals a hypervigilance to threatening stimuli. Surprisingly, this hypervigilance is unaffected by mother or child mental health, suggesting that other potential cognitive mechanisms of intergenerational psychopathology transmission should be explored in the refugee context with complex trauma exposure.

### Electronic Supplementary Material

Below is the link to the electronic supplementary material.

Supplementary Material 1
