# Supplementary material for: Maternal Trauma and Psychopathology Symptoms Affect Refugee Children’s Mental Health But Not Their Emotion Processing
Source: Res Child Adolesc Psychopathol. 2024 Mar 2;52(8):1233–46. doi: 10.1007/s10802-024-01182-0 (PMC11289056; doi:10.1007/s10802-024-01182-0)

# Supporting Information

**Appendix S1. Data collection information**

| **Measure** | **T1** | | **T2** | |
| --- | --- | --- | --- | --- |
|  | Child | Mother | Child | Mother |
| Attention bias task | **x** |  |  | **x** |
| Child mental health |  |  |  | **x** |
| Household wealth |  | **x** |  |  |
| Maternal trauma exposure |  |  |  | **x** |
| Maternal PTSD symptoms |  |  |  | **x** |
| Maternal anxiety symptoms |  |  |  | **x** |
| Maternal depression symptoms |  |  |  | **x** |

T1: February-May 2021; T2: May-August 2021

**Appendix S2. Trauma and mental health questionnaires details**

**Maternal trauma and mental health**

*Trauma*

Maternal trauma was measured with the self-reported Traumatic Events Checklist (TEC, Panter-Brick et al., 2009), consisting of 21 *yes/no* questions related to war trauma and displacement. Items included events such as having lived in a refugee camp and having witnessed bombardment (see Figure S1 for all items and response breakdown). Due to the distressing nature of the trauma questionnaire, only a subset (*n* = 140) of participating mothers completed this measure before it was replaced with a less distressing PTSD questionnaire.

*Symptoms of Post-Traumatic Stress Disorder (PTSD)*

Maternal PTSD symptoms were assessed with PTSD Checklist for DSM-5 (PCL-5, Weathers et al., 2013) , which includes 20 items (Cronbach’s α = .92). This scale was completed by a subset of participants (*n* = 129), tested from June onwards. PCL-5 is a 5-point Likert scale with responses ranging from 0 (*not at all*) to 4 (*extremely*) to a list of problems that people might have in response to a very stressful experience. The participating mothers were asked to keep their worst event in mind and indicate how much they have been bothered by each problem in the past month, with questions such as ‘Repeated, disturbing, and unwanted memories of the stressful experience?’ and ‘Blaming yourself or someone else for the stressful experience or what happened after it?’. A total symptom severity score is calculated by summing all scores and can range from 0 to 80, with scores over 31-33 indicative of probable PTSD (Weathers et al., 2013).

*Symptoms of anxiety*

The anxiety subscale of the short form Depression, Anxiety, Stress (DASS-21, Henry & Crawford, 2005) was used to measure maternal anxiety symptoms (7 items, Cronbach’s α = .79). DASS-A is a 4-point Likert-scale ranging from 0 (*did not apply to me - never*) to 3 (*applied to me very much, or most of the time – almost always*) which includes statements, such as ‘I felt scared without any good reason’ and ‘I was aware of dryness in my mouth’. Participating mothers were asked to what extent each statement applies to their feelings over the past month. Scores between 10 and 19 are indicative of moderate to severe anxiety (Henry & Crawford, 2005).

*Symptoms of depression*

Maternal depression symptoms were measured using the Centre for Epidemiological Studies – Depression Scale (CES-D, Radloff, 1977), which includes 10 items (Cronbach’s α = .79) on a 4-point Likert scale ranging from 0 (*rarely, or none of the time*) to 3 (*most, or almost all the time*). Participating mothers were asked about the way they have felt or behaved over the past month, with statements such as ‘I felt depressed (inner sadness)’ and ‘I felt that everything I did was an effort’. Two items (‘I felt hopeful about the future’ and ‘I was happy’) were reverse-scored and depression symptoms score was obtained by summing all items, with higher score indicative of higher symptoms severity, with scores above 10 suggesting potential depression (Radloff, 1977).

**Child mental health**

All child measures were reported by their mothers, using the short version of the Paediatric Symptoms Checklist (PCS – 17, Jellinek et al., 1988). PCS-17 contains three subscales, which measure separate constructs: internalising, externalising, and attention problems, on a 3-point Likert scale ranging from 0 (*never*) to 2 (*often*). Internalising subscale (5 items, Cronbach’s α = .59) contains statements related to the child’s feelings of anxiety and depression, such as ‘Feels sad, unhappy’ and ‘Worries a lot’. Externalising subscale (7 items, Cronbach’s α = .74) includes statements related to the child’s conduct and behavioural problems, such as ‘Fights with other children’ and ‘Does not listen to rules’. Attention subscale (5 item, Cronbach’s α = .65) relates to child’s inattention and hyperactivity, e.g., ‘Distracted easily’ and ‘Fidgety, unable to sit still’.


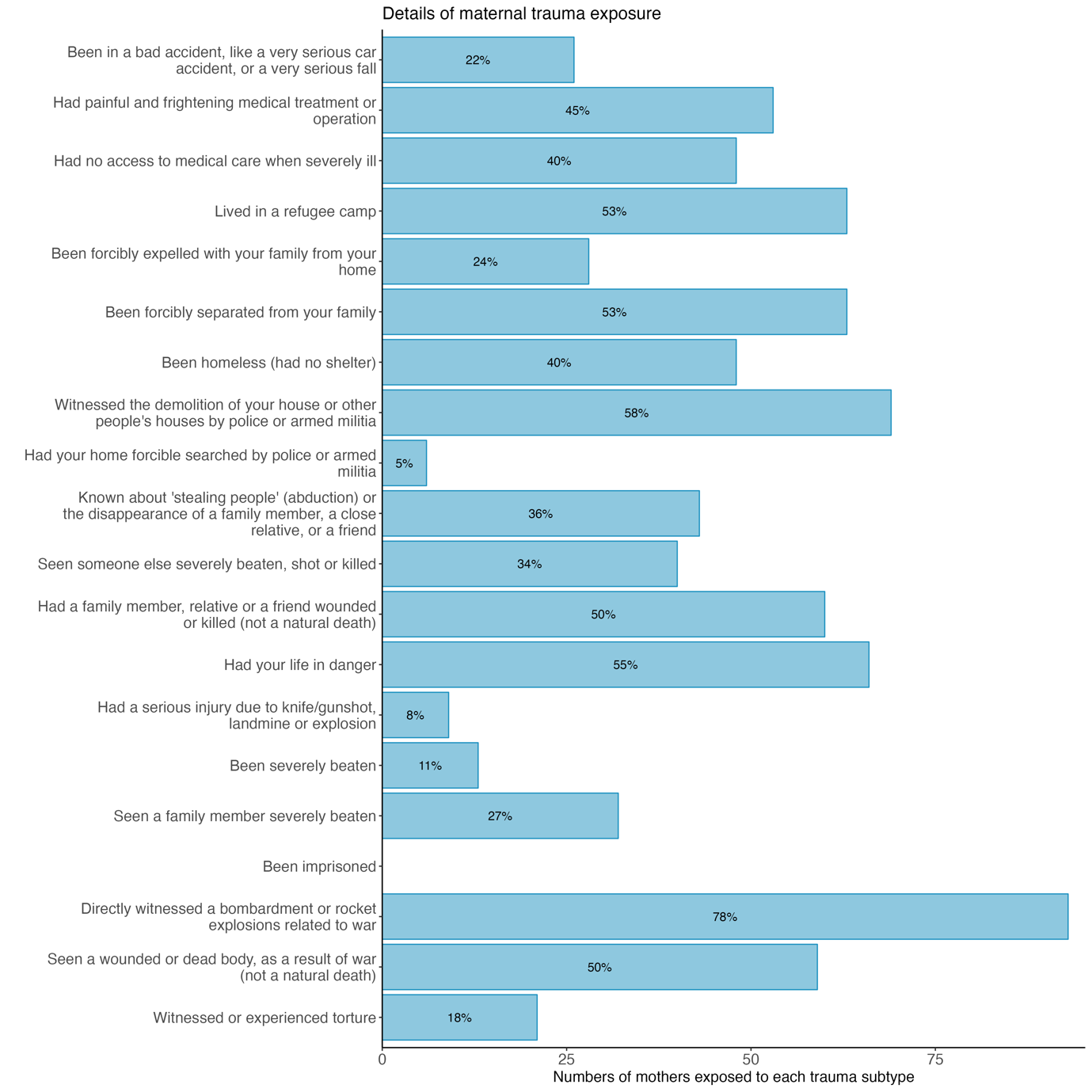


**Figure S1. Details of the traumatic events experienced by the mothers (TEC questions).**

**Table S1. Demographics and mental health information of the whole sample.**

| **Variable** | ***Scale range*** | ***N*** | ***M*** | ***SD*** |
| --- | --- | --- | --- | --- |
| **Children (boys *n* = 165, 50%)** | | | | |
| Age |  | 321 | 6.32 | 1.18 |
| Born in Jordan |  | 261 (82%) |  |  |
| Internalising problems | 0-10 | 294 | 3.21 | 2.02 |
| Externalising problems | 0-14 | 291 | 5.09 | 3.06 |
| Attention problems | 0-10 | 293 | 5.19 | 2.38 |
| **Mothers** | | | | |
| Age |  | 320 | 32.61 | 7.02 |
| Household wealth |  | 317 | 7.92 | 1.89 |
| Time spent in Jordan (years) |  | 318 | 8.31 | 2.51 |
| Lives in Amman |  | 235 (73%) |  |  |
| Trauma exposure | 0-21 | 133 | 7.52 | 4.69 |
| PSTD symptoms | 0-80 | 124 | 34.96 | 16.24 |
| Anxiety symptoms | 0-21 | 289 | 6.60 | 4.60 |
| Depression symptoms | 0-30 | 284 | 13.95 | 6.27 |

*M* = mean, *SD* = standard deviation. Trauma = TEC (Traumatic Events Checklist), PTSD symptoms = PCL-5 (PTSD Checklist for DSM-5), Anxiety symptoms = DASS-Anxiety (Anxiety subscale of the Depression, Anxiety, Stress Scale short form), Depression symptoms = (CES-Depression (Centre for Epidemiological Studies – Depression Scale), PSC-17 = Paediatric Symptoms Checklist (child internalising, externalising, and attention problems).

**Table S2. Demographics and mental health information of participants who took part in the dot probe experiment.**

| **Variable** | ***Scale range*** | ***N*** | ***M*** | ***SD*** |
| --- | --- | --- | --- | --- |
| **Children *n* = 264 (boys *n* = 129, 49%)** | | | | |
| Age |  | 261 | 6.49 | 1.13 |
| Born in Jordan |  | 209 (79%) |  |  |
| Internalising problems | 0-10 | 239 | 3.28 | 2.02 |
| Externalising problems | 0-14 | 238 | 5.12 | 3.03 |
| Attention problems | 0-10 | 238 | 5.13 | 2.34 |
| **Mothers *n* = 298** | | | | |
| Age |  | 296 | 32.47 | 6.98 |
| Household wealth |  | 292 | 7.93 | 1.83 |
| Time spent in Jordan (years) |  | 295 | 8.26 | 2.24 |
| Lives in Amman |  | 216 (73%) |  |  |
| Trauma exposure | 0-21 | 127 | 7.47 | 4.73 |
| PSTD symptoms | 0-80 | 119 | 35.18 | 16.29 |
| Anxiety symptoms | 0-21 | 274 | 6.61 | 4.61 |
| Depression symptoms | 0-30 | 271 | 14.00 | 6.23 |

**Table S3.**

*Associations between maternal trauma and mental health measures.*

| *Variable* | 1 | 2 | 3 | 4 |
| --- | --- | --- | --- | --- |
| 1. TEC | - |  |  |  |
| 2. PCL-5 | - | - |  |  |
| 3. DASS-A | .34 | .56 | - |  |
| 4. CES-D | .43 | .70 | .64 | - |

*Note.* All correlations are significant at *p* < .01. Participants completed either TEC or PCL-5 measure, never both; hence, these two variables cannot be correlated.

**Table S4.**

*Reaction times in seconds for each emotion pair, separately for congruent and incongruent trials.*

| *Participants* | Emotion type | Condition | *M* | *SD* |
| --- | --- | --- | --- | --- |
| Children | Angry - neutral | congruent | 1.07 | .43 |
|  |  | incongruent | 1.11 | .46 |
|  | Sad - neutral | congruent | 1.09 | .43 |
|  |  | incongruent | 1.10 | .44 |
|  | Baseline (neutral – neutral) | | 1.09 | .47 |
| Mothers | Angry - neutral | congruent | .56 | .16 |
|  |  | incongruent | .58 | .16 |
|  | Sad - neutral | congruent | .56 | .15 |
|  |  | incongruent | .56 | .15 |
|  | Baseline (neutral – neutral) | | .57 | .17 |

**Table S5.**

*Linear regression models of the effects of maternal trauma and psychopathology symptoms on child and mother attention biases.*

| *Mothers’ outcomes* | *F* | *df* | *p* | *adj.R^2^* | *t* | *b* | | *β* |
| --- | --- | --- | --- | --- | --- | --- | --- | --- |
| **Child attention biases** | | | | | | | | |
| *Anger bias* | | | | | | | | |
| Trauma exposure | 0.07 | 1, 107 | .797 | -.009 | -0.26 | -<.001 | -.03 | |
| PTSD | 0.42 | 1, 92 | .521 | -.006 | -0.64 | -.001 | -.07 | |
| Anxiety | 1.54 | 1, 227 | .217 | .002 | 1.24 | .003 | .08 | |
| Depression | 2.46 | 1, 220 | .118 | .007 | 1.57 | .003 | .11 | |
| *Sadness bias* | | | | | | | | |
| Trauma exposure | 0.27 | 1, 110 | .605 | -.006 | -0.52 | -.002 | -.05 | |
| PTSD | 2.13 | 1, 94 | .147 | .001 | -1.46 | -.001 | -.15 | |
| Anxiety | 2.94 | 1, 231 | .088 | .008 | -1.71 | -.004 | -.11 | |
| Depression | 2.16 | 1, 224 | .143 | -.005 | -1.47 | -.002 | -.10 | |
| **Mother attention biases** | | | | | | | | |
| *Anger bias* | | | | | | | | |
| Trauma exposure | 1.38 | 1, 124 | .003 | .003 | -1.76 | -.001 | -.11 | |
| PTSD | 0.14 | 1, 116 | .707 | -.007 | 0.38 | <.001 | .04 | |
| Anxiety | 1.06 | 1, 269 | .304 | <.001 | 1.03 | .001 | .06 | |
| Depression | 1.78 | 1, 266 | .183 | .003 | 1.334 | .001 | .08 | |
| *Sadness bias* | | | | | | | | |
| Trauma exposure | 0.60 | 1, 124 | .442 | -.003 | -0.77 | -.001 | -.07 | |
| PTSD | 1.88 | 1, 117 | .173 | .007 | -1.37 | <.001 | -.13 | |
| Anxiety | 0.18 | 1, 268 | .669 | -.003 | 0.43 | <.001 | .03 | |
| Depression | 0.03 | 1, 265 | .869 | -.004 | -0.17 | <-.001 | <-.01 | |

**Table S6.**

*Linear regression models of the effects of child internalising, externalising, and attention problems on their attention biases*

| *Predictors* | *Estimates* | *CI* | *p* |
| --- | --- | --- | --- |
| *Anger bias (n = 228)* | | | |
| (Intercept) | 0.04 | -0.01 – 0.10 | 0.083 |
| Internalising problems | -0.00 | -0.01 – 0.01 | 0.791 |
| Externalising problems | 0.01 | -0.00 – 0.01 | 0.150 |
| Attention problems | -0.01 | -0.02 – 0.00 | 0.100 |
| R^2^ / R^2^ adjusted: 0.017 / 0.004 |  |  |  |
| *Sadness bias (n = 232)* | | | |
| (Intercept) | 0.02 | -0.03 – 0.07 | 0.354 |
| Internalising problems | -0.00 | -0.01 – 0.01 | 0.935 |
| Externalising problems | 0.00 | -0.01 – 0.01 | 0.989 |
| Attention problems | -0.00 | -0.01 – 0.01 | 0.541 |
| R^2^ / R^2^ adjusted: 0.003 / -0.011 |  |  |  |

**Table S7.**

*Control and experimental group comparisons for all variables of interest*

|  | *t* | *df* | *p* | *d* | *95% CI* |
| --- | --- | --- | --- | --- | --- |
| *Child measures* | | | | | |
| Anger bias | 0.97 | 244.27 | .34 | 0.12 | -0.02 – 0.05 |
| Sadness bias | 0.20 | 245.48 | .84 | 0.03 | -0.03 – 0.04 |
| Internalising symptoms | -0.15 | 288.39 | .89 | -0.02 | -0.50 – 0.43 |
| Externalising symptoms | -0.89 | 281.76 | .38 | -0.10 | -1.03 – 0.39 |
| Attention symptoms | -0.33 | 287.39 | .74 | -0.04 | -0.64 – 0.46 |
| *Maternal measures* | | | | | |
| Anger bias | 1.28 | 285.18 | .20 | 0.15 | -0.00 – 0.02 |
| Sadness bias | 0.55 | 262.28 | .58 | 0.06 | -0.01 – 0.01 |
| Depression symptoms | -0.68 | 280.70 | .50 | -0.08 | -1.98 – 0.96 |
| Anxiety symptoms | -1.63 | 285,83 | .10 | -0.19 | -1.94 – 0.18 |
| PTSD symptoms | 0.65 | 121.96 | .52 | 0.12 | -3.90 – 7.67 |
| Trauma exposure | -3.91 | 129.72 | <.001 | -0.68 | -4.56 - -1.50 |

**Table S8.**

*Linear regression models of the effects of maternal trauma and psychopathology symptoms on child and mother attention biases controlling for reading programme participation.*

| *Maternal predictors* | *Estimates* | *CI* | *p* | *N* | R^2^ / R^2^ adj |
| --- | --- | --- | --- | --- | --- |
| *Child anger bias* | | | | | |
| Trauma exposure | 0.00 | -0.01 – 0.01 | 0.983 | 108 | 0.006 / -0.013 |
| Group | -0.02 | -0.08 – 0.04 | 0.452 |  |  |
| PTSD symptoms | -0.00 | -0.00 – 0.00 | 0.533 | 94 | 0.005 / -0.017 |
| Group | 0.00 | -0.06 – 0.06 | 0.949 |  |  |
| Anxiety symptoms | 0.00 | -0.00 – 0.01 | 0.175 | 228 | 0.012 / 0.004 |
| Group | -0.02 | -0.06 – 0.02 | 0.286 |  |  |
| Depression symptoms | 0.00 | -0.00 – 0.01 | 0.112 | 221 | 0.016 / 0.007 |
| Group | -0.02 | -0.06 – 0.02 | 0.301 |  |  |
| *Child sad bias* | | | | | |
| Trauma exposure | -0.00 | -0.01 – 0.00 | 0.631 | 111 | 0.002 / -0.016 |
| Group | -0.00 | -0.06 – 0.06 | 0.952 |  |  |
| PTSD symptoms | -0.00 | -0.00 – 0.00 | 0.132 | 96 | 0.028 / 0.007 |
| Group | -0.02 | -0.07 – 0.03 | 0.475 |  |  |
| Anxiety symptoms | -0.00 | -0.01 – 0.00 | 0.091 | 232 | 0.013 / 0.004 |
| Group | -0.00 | -0.04 – 0.04 | 0.948 |  |  |
| Depression symptoms | -0.00 | -0.01 – 0.00 | 0.147 | 225 | 0.010 / 0.001 |
| Trauma exposure | -0.01 | -0.04 – 0.03 | 0.776 |  |  |
| *Maternal anger bias* | | | | | |
| Trauma exposure | -0.00 | -0.00 – 0.00 | 0.485 | 125 | 0.019 / 0.003 |
| Group | -0.01 | -0.02 – 0.01 | 0.299 |  |  |
| PTSD symptoms | 0.00 | -0.00 – 0.00 | 0.712 |  | 0.001 / -0.016 |
| Group | -0.00 | -0.01 – 0.01 | 0.974 | 118 |  |
| Anxiety symptoms | 0.00 | -0.00 – 0.00 | 0.233 | 270 | 0.011 / 0.003 |
| Group | -0.01 | -0.02 – 0.00 | 0.190 |  |  |
| Depression symptoms | 0.00 | -0.00 – 0.00 | 0.167 | 267 | 0.014 / 0.006 |
| Trauma exposure | -0.01 | -0.02 – 0.00 | 0.175 |  |  |
| *Maternal sadness bias* | | | | | |
| Trauma exposure | -0.00 | -0.00 – 0.00 | 0.254 | 125 | 0.018 / 0.002 |
| Group | 0.01 | -0.01 – 0.03 | 0.186 |  |  |
| PTSD symptoms | -0.00 | -0.00 – 0.00 | 0.161 |  | 0.020 / 0.004 |
| Group | -0.01 | -0.02 – 0.01 | 0.462 | 119 |  |
| Anxiety symptoms | 0.00 | -0.00 – 0.00 | 0.622 | 269 | 0.001 / -0.007 |
| Group | -0.00 | -0.01 – 0.01 | 0.903 |  |  |
| Depression symptoms | -0.00 | -0.00 – 0.00 | 0.889 | 266 | 0.000 / -0.007 |
| Trauma exposure | -0.00 | -0.01 – 0.01 | 0.799 |  |  |

**Table S9.**

*Linear regression models of the effects of maternal trauma and psychopathology symptoms on child mental health controlling for reading programme participation.*

| *Maternal predictors* | *Estimates* | *CI* | *p* | *N* | R^2^ / R^2^ adj |
| --- | --- | --- | --- | --- | --- |
| *Child internalising problems* | | | | | |
| Trauma exposure | 0.16 | 0.08 – 0.24 | **<0.001** | 132 | 0.118 / 0.104 |
| Group | -0.65 | -1.38 – 0.07 | 0.076 |  |  |
| PTSD symptoms | 0.03 | 0.01 – 0.05 | **0.002** | 123 | 0.090 / 0.075 |
| Group | 0.51 | -0.15 – 1.18 | 0.127 |  |  |
| Anxiety symptoms | 0.10 | 0.05 – 0.15 | **<0.001** | 285 | 0.050 / 0.043 |
| Group | -0.07 | -0.53 – 0.40 | 0.779 |  |  |
| Depression symptoms | 0.13 | 0.09 – 0.16 | **<0.001** | 280 | 0.162 / 0.156 |
| Group | 0.05 | -0.39 – 0.48 | 0.830 |  |  |
| *Child externalising problems* | | | | | |
| Trauma exposure | 0.04 | -0.07 – 0.16 | 0.480 | 129 | 0.009 / -0.007 |
| Group | 0.29 | -0.81 – 1.38 | 0.603 |  |  |
| PTSD symptoms | 0.03 | 0.00 – 0.06 | **0.044** | 124 | 0.033 / 0.017 |
| Group | 0.14 | -0.89 – 1.18 | 0.785 |  |  |
| Anxiety symptoms | 0.17 | 0.10 – 0.25 | **<0.001** | 282 | 0.071 / 0.064 |
| Group | 0.15 | -0.55 – 0.85 | 0.676 |  |  |
| Depression symptoms | 0.12 | 0.07 – 0.18 | **<0.001** | 277 | 0.067 / 0.061 |
| Group | 0.32 | -0.39 – 1.02 | 0.376 |  |  |
| *Child attention problems* | | | | | |
| Trauma exposure | 0.10 | 0.01 – 0.19 | **0.023** | 132 | 0.052 / 0.037 |
| Group | 0.21 | -0.61 – 1.03 | 0.617 |  |  |
| PTSD symptoms | 0.05 | 0.02 – 0.07 | **<0.001** |  | 0.120 / 0.105 |
| Group | -0.33 | -1.14 – 0.48 | 0.425 | 122 |  |
| Anxiety symptoms | 0.12 | 0.06 – 0.18 | **<0.001** | 284 | 0.057 / 0.051 |
| Group | -0.03 | -0.57 – 0.51 | 0.922 |  |  |
| Depression symptoms | 0.11 | 0.07 – 0.15 | **<0.001** | 280 | 0.088 / 0.081 |
| Group | 0.08 | -0.45 – 0.62 | 0.758 |  |  |

**Table S10.**

*Linear regression models of the effects of child internalising, externalising, and attention problems on their attention biases controlling for reading programme participation.*

| *Predictors* | *Estimates* | *CI* | *p* |
| --- | --- | --- | --- |
| *Anger bias (n = 227)* | | | |
| (Intercept) | 0.06 | 0.00 – 0.11 | **0.047** |
| Internalising problems | -0.00 | -0.01 – 0.01 | 0.736 |
| Externalising problems | 0.01 | -0.00 – 0.01 | 0.130 |
| Attention problems | -0.01 | -0.02 – 0.00 | 0.088 |
| Group* | -0.02 | -0.06 – 0.02 | 0.304 |
| R^2^ / R^2^ adjusted: 0.023 / 0.005 |  |  |  |
| *Sadness bias (n = 231)* | | | |
| (Intercept) | 0.03 | -0.03 – 0.08 | 0.339 |
| Internalising problems | -0.00 | -0.01 – 0.01 | 0.921 |
| Externalising problems | 0.00 | -0.01 – 0.01 | 0.978 |
| Attention problems | -0.00 | -0.01 – 0.01 | 0.533 |
| Group* | -0.01 | -0.04 – 0.03 | 0.771 |
| R^2^ / R^2^ adjusted: 0.003 / -0.015 |  |  |  |

*Note.* *Group = reading programme participation (control, experimental). CI = 95% Confidence Intervals

**Table S11.**

*Linear regression models of the association between mother-child attentional biases controlling for reading programme participation.*

| *Predictors* | *Estimates* | *CI* | *p* |
| --- | --- | --- | --- |
| *Child anger bias (n = 231)* | | | |
| (Intercept) | 0.03 | 0.00 – 0.06 | **0.050** |
| Maternal anger bias | 0.20 | -0.26 – 0.65 | 0.394 |
| Group | -0.01 | -0.04 – 0.03 | 0.745 |
| R^2^ / R^2^ adjusted: 0.004 / -0.005 |  |  |  |
| *Child sadness bias (n = 232)* | | | |
| (Intercept) | 0.01 | -0.01 – 0.04 | 0.352 |
| Maternal sadness bias | 0.03 | -0.44 – 0.50 | 0.899 |
| Group | -0.01 | -0.04 – 0.03 | 0.714 |
| R^2^ / R^2^ adjusted: 0.001 / -0.008 |  |  |  |

**Figure S2.**

*Associations between mother-child mental health and trauma outcomes*

*
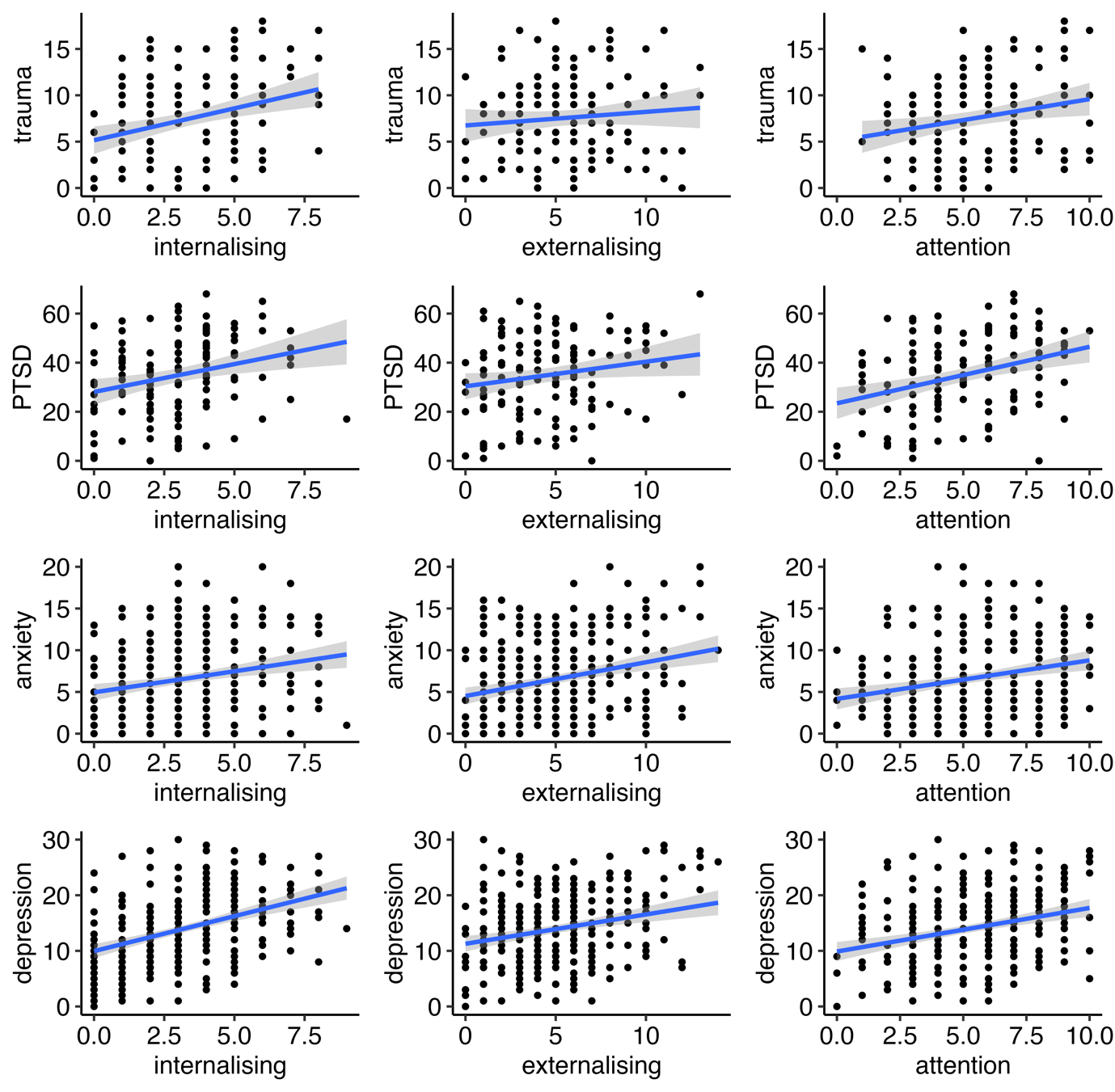
*

*Note.* Child internalising, externalising, and attention problems (PSC-17) on the x axis. Mothers’ trauma exposure and symptoms of PTSD, anxiety, and depression on the y axis.

**Figure S3.**

*Associations between child and mother attention biases to angry and sad expressions.*


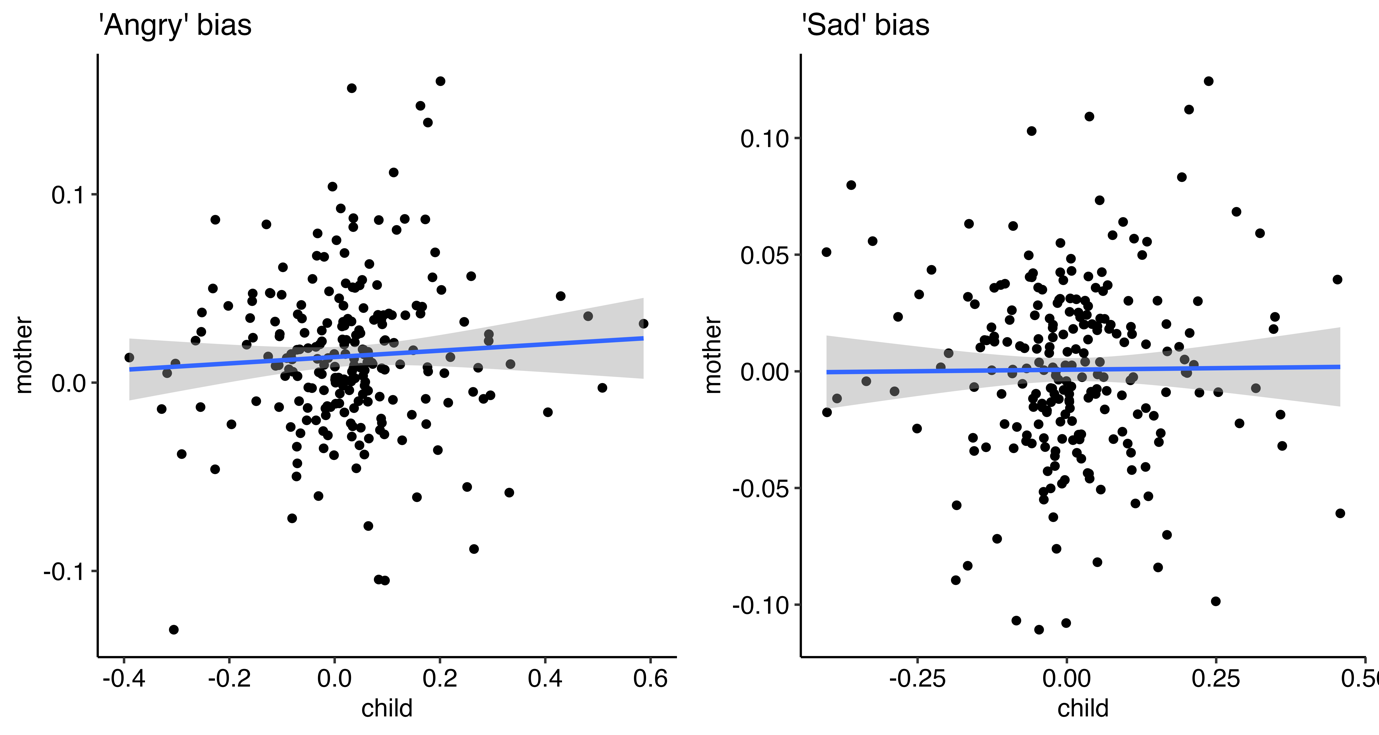

Supplement: Supplementary file 1 — Supplementary Material 1 [file 10802_2024_1182_MOESM1_ESM.docx]
